# Should Postoperative Radiation for Long Bone Metastases Cover Part or All of the Orthopedic Hardware? Results of a Large Retrospective Analysis

**DOI:** 10.1016/j.adro.2021.100756

**Published:** 2021-07-28

**Authors:** Daniel B. Rosen, Justin M. Haseltine, Meredith Bartelstein, Jessica R. Flynn, Zhigang Zhang, Zachary A. Kohutek, Yoshiya Yamada, Adam Schmitt, Daniel S. Higginson, Maksim Vaynrub, Jonathan T. Yang, Erin F. Gillespie

**Affiliations:** a Department of Radiation Oncology, Memorial Sloan Kettering Cancer Center, New York, New York; b Orthopaedic Service, Department of Surgery, Memorial Sloan Kettering Cancer Center, New York, New York; c Department of Epidemiology & Biostatistics, Memorial Sloan Kettering Cancer Center, New York, New York; d Vanderbilt-Ingram Cancer Center, Nashville, Tennessee

## Abstract

**Purpose:**

For patients with long bone metastases who undergo orthopedic stabilization surgery followed by radiotherapy (RT), it is unclear what extent of hardware coverage by the radiation field is needed for optimal tumor control.

**Methods and Materials:**

Long bone metastases treated with surgical intervention followed by radiation between August 2011 to May 2019 from a single institution were reviewed. Local recurrence, defined as any in-bone recurrence, was identified by chart review. Accompanying demographic and treatment characteristics were recorded. Statistical analysis to evaluate factors associated with tumor recurrence included univariate analysis, multivariate analysis, and propensity score matching.

**Results:**

Among 138 patients with 145 long bone metastases undergoing postoperative RT with a median follow-up of 29.5 months, 36 bone metastases experienced a local recurrence. Most patients (92%) were treated with conventional RT and the median delivered dose was 30 Gy (interquarile range, 20-30 Gy). On univariate analysis, whole hardware RT field coverage and higher dose (biologically effective dose 10 ≥39 Gy) were associated with reduced local recurrence (0.44 hazard ratio [HR]; 95% confidence interval [CI], 0.22%-0.86%; *P* = .017; 0.5 HR; 95% CI, 0.26%-0.96%; *P* = .038, respectively). Covariates of time from surgery to RT start, histology of primary tumor (categorized as resistant vs sensitive), intramedullary hardware placement, reaming procedure, and margin status did not reach statistical significance. To adjust for confounding effects, we also conducted a propensity score matched analysis which confirmed that whole hardware coverage was statistically associated with a decreased risk of recurrence on the matched dataset (0.24 HR; 95% CI, 0.07%-0.84%; *P* = .026).

**Conclusions:**

In this analysis of mostly patients undergoing conventional radiation, coverage of the whole hardware was associated with reduced local recurrence for patients with long bone metastases, consistent with prior reports. Investigation of approaches to further reduce local recurrence, such as preoperative stereotactic radiation, may be warranted.

## Introduction

Long bone osseous metastases are common, occurring in up to 75% of patients with metastatic breast and prostate cancer and can cause instability or pathologic fracture requiring surgical stabilization to facilitate limb use and improve pain control.^1^ Radiotherapy (RT) after orthopedic stabilization significantly improves functional status and reduces hardware failure.^2^ Postoperative RT has thus become the standard of care after stabilization of long-bone metastases.

The optimal radiation field design after stabilization of long bone metastases remains a point of controversy. The aforementioned landmark retrospective study published in 1994 reported that 84% (21/25) of evaluable patients involved radiation fields covering the entire orthopedic hardware.^2^ This approach reflects the concern that surgical stabilization, particularly intramedullary instrumentation, may lead to distal seeding of tumor within the long bone.^3^ Common surgical techniques, such as intramedullary reaming, may further affect outcomes. Subsequent reports have continued to support the practice of greater orthopedic hardware coverage, although they are small and include spinal metastases^3^ as well as nonsolid tumor malignancies.^4^ As a result, clinical practice continues to be heterogeneous.

In this study, we evaluated the largest modern cohort of patients with non-spine long bone metastases for factors associated with recurrence after orthopedic stabilization, with special focus on extent of orthopedic hardware coverage by the radiation field.

## Methods and Materials

### Study sample and follow-up

Patients with metastatic lesions in long bones from solid tumors managed with orthopedic stabilization followed by RT from August 1, 2011, to May 9, 2019 were retrospectively reviewed in accordance with institutional ethical practices. Patients were eligible if they underwent RT within 4 months after orthopedic stabilization and had follow-up imaging available at least 3 months after RT completion. Patients with prior RT or surgical intervention to the bone were excluded. Usual follow-up included a clinic visit every 3 to 6 months with concurrent routine imaging. Patient medical records including diagnostic images were reviewed (J.M.H. and D.B.R.) to confirm inclusion/exclusion criteria and record endpoints and covariates.

### Surgical treatment

In general, surgical stabilization was performed for long bones at risk for pathologic fracture or with existing fracture due to metastatic disease. Tumor excision, intraoperative adjuvant treatment, and surgical stabilization were performed at the discretion of the treating surgeon. Retrospective chart review was performed by an orthopedic surgeon (M.B.) to determine extent of excision, class of orthopedic implant, use of intraoperative adjuvant treatment, and other relevant factors. Classes of hardware included intramedullary nail, endoprosthesis, and plate. Hardware types were coded as intramedullary if the hardware traversed the intramedullary cavity along the longitudinal axis (eg, intramedullary nail, endoprosthesis, intercalary prosthesis, dynamic hip screws) or nonintramedullary if the hardware entered perpendicular to the cortex (ie, plate fixation). Procedures were either performed for the purpose of stabilization alone or in conjunction with tumor excision prior to hardware implantation.

### Radiation treatment

After orthopedic stabilization, patients underwent radiation treatment to the bone with dose, fractionation, technique, and extent of target volume coverage determined by the treating physician. Time from orthopedic stabilization to RT was dependent upon clinical factors such as wound healing and was minimized to the extent possible. Conventionally-fractionated radiation was administered daily using AP/PA fields with dose prescribed to midplane. SBRT was administered daily or every other day using either step-and-shot or volumetric arc techniques with dose prescribed to the PTV. During the study period, there was no institutional policy to guide extent of target volume in this setting and practice patterns varied.

### Outcome measures and covariates

The primary endpoint was local recurrence with the competing risk of patient death. Local recurrence was defined as any in-bone recurrence, including those in-field and out-of-field, after the completion of RT. In-field recurrence was defined as a recurrence that completely overlapped with the RT field, marginal recurrence was a recurrence that partially overlapped the RT field, and out-of-field recurrence was a recurrence that had no overlap with the RT field. Medical physician notes as well as primary radiographs from x-rays, computed tomography, magnetic resonance imaging, and positron emission tomography modalities were reviewed to determine recurrence date (if present) and location compared to the treatment field. Discrepancies in data collection were reviewed by 4 authors (J.M.H., D.B.R., E.F.G., M.B.) including one attending radiation oncology physician and one attending surgeon.

The major covariate of interest was the extent of RT target coverage, which we defined as a binary variable with RT field measuring the full length of the orthopedic hardware (whole coverage) versus RT field measuring less than the full length of the hardware (partial coverage). Other covariates included age at surgery, sex, radioresistant, or nonradioresistant primary histology (as previously defined by Laufer et al^5^), time (days) from surgery to radiation therapy start, Karnofsky Performance Status (KPS), occurrence of reaming procedure during orthopedic stabilization, margin status (defined as en bloc resection with negative margins versus en bloc resection with positive margins or no en bloc resection), hardware type (intramedullary defined by orthopedic hardware disrupting the intramedullary cavity along its longitudinal axis ie, intramedullary nails, endoprostheses, intercalary devices but not plates), and biologically effective dose at an α/β ratio of 10 (BED10).^6^ Additionally, a novel covariate, translesional status, was recorded. Surgical treatment was determined to be translesional if metastatic resection did not occur (or resulted in a positive margin) and the implanted hardware was of the intramedullary type, suggesting that surgical seeding of the medullary canal had occurred. If the margins were negative or if the hardware was not intramedullary, the treatment was recorded as nontranslesional.

### Statistical methods

In patients with more than one bone treated with orthopedic stabilization and RT, each bone was analyzed as independently. Descriptive statistics were reported by field coverage using median and interquartile range for continuous variables and proportions for categorical variables. Univariate analysis for tumor recurrence was conducted using fine gray models with a competing risk of death. The covariates BED10 and KPS were dichotomized based on median values. Covariates meeting the prescribed significance threshold (α = 0.05), were further analyzed in a multivariable model. Fisher exact tests were used for examining correlation between covariates. A propensity score match was used based on a logistic regression model with the outcome of coverage and covariates age, sex, primary histology, time to RT start, KPS, reaming operation, translesional status, margins status, BED10, and hardware. Univariate analysis for the endpoint of tumor recurrence and the covariate of coverage was conducted on the matched dataset. All analyses were performed using R, version 3.6.2.

## Results

### Patient characteristics

One hundred thirty-eight patients with 145 metastatic tumors to bone met inclusion criteria. Seventy metastatic tumors (48%) were treated with radiation to part of the implanted orthopedic hardware while 75 lesions (52%) were treated with RT covering the whole length of the implanted hardware. Table 1 shows baseline characteristics for each tumor in the 2 groups. Patients receiving whole hardware RT coverage were less likely to have undergone a reaming procedure, more likely to have a BED10 greater than or equal to the median of 39 Gy, and less likely to have hardware traversing the intramedullary cavity of the bone. Twelve out of 145 lesions (8%) were treated with SBRT. The most common primary tumor sites were renal (30%, n = 43), breast (25%, n = 36), and lung (17%, n = 24).

**Table 1:** Baseline characteristics of bone metastases undergoing postoperative radiation

Demographic		Total		Partial		Whole		
		n =	(percent)	n =	(percent)	n =	(percent)	*P* value
Total		145		70	(48%)	75	(52%)	
Age*	(y)	63	(54-69)	64	(55-70)	62	(54-68)	.3
Sex								>.9
	Female	81	(56%)	39	(56%)	42	(56%)	
	Male	64	(44%)	31	(44%)	33	(44%)	
Primary histology								.7
	Nonresistant	63	(43%)	29	(41%)	34	(45%)	
	Resistant	82	(57%)	41	(59%)	41	(55%)	
Time to RT start*	(d)	40	(30-50)	35	(26-49)	41	(35-54)	.019
KPS*		80	(70-80)	80	(70-80)	80	(70-80)	.14
	≥ Median (80)	71	(49%)	29	(41%)	42	(56%)	
	< Median (80)	49	(34%)	27	(39%)	22	(29%)	
	Not recorded	25	(17%)	14	(20%)	11	(15%)	
Reaming								.003
	Yes	118	(81%)	64	(91%)	54	(72%)	
	No	27	(19%)	6	(9%)	21	(28%)	
Translesional								.049
	Yes	111	(77%)	59	(84%)	52	(69%)	
	No	34	(23%)	11	(16%)	23	(31%)	
Margins								>.9
	Negative	15	(10%)	7	(10%)	8	(11%)	
	Positive or none	130	(90%)	63	(90%)	67	(89%)	
BED10*	(Gy)	39	(28-39)	37.5	(28-39)	39	(28-39)	.004
	≥ Median (39)	84	(58%)	32	(46%)	52	(69%)	
	< Median (39)	61	(42%)	38	(54%)	23	(31%)	
Hardware								.009
	Intramedullary	112	(77%)	61	(87%)	51	(68%)	
	Plate	33	(23%)	9	(13%)	24	(32%)	

⁎ Statistics presented: median (interquartile range). *Abbreviations*: BED10 = biologically effective dose (*α*/*β* = 10); KPS = Karnofsky Performance Status; RT = radiotherapy.

### Tumor recurrence

The median follow-up of the cohort was 30 months, and 36 metastatic tumors experienced a local recurrence. The cumulative incidence of recurrence at median follow-up was 30% (95% confidence interval [CI], 22%-39%). The 1-year local recurrence rate was 12% (95% CI, 6.0%-21%) for whole hardware coverage and 21% (95% CI, 12%-32%) for partial coverage. The 2-year local recurrence rate increased to 16% (95% CI, 8.3%-26%) for whole coverage and 41% (95% CI, 27%-54%) for partial coverage. There were 17 hardware failure events, 11 of which (65%) were related to tumor recurrence.

Of the 36 recurrences, 9 (25%) were out of field. The partial hardware treatment group accounted for 24 recurrences, of which 7 (29%) were out of field. The whole hardware treatment group accounted for 12 recurrences, of which 2 (16%) were out of field. The partial hardware group appeared to result in more out-of-field recurrences than the whole hardware group. However, we deferred inferential statistics on the subset analysis of in-field versus out-of-field recurrence owing to the relatively small number of recurrences and variable follow-up.

**Figure 1:**
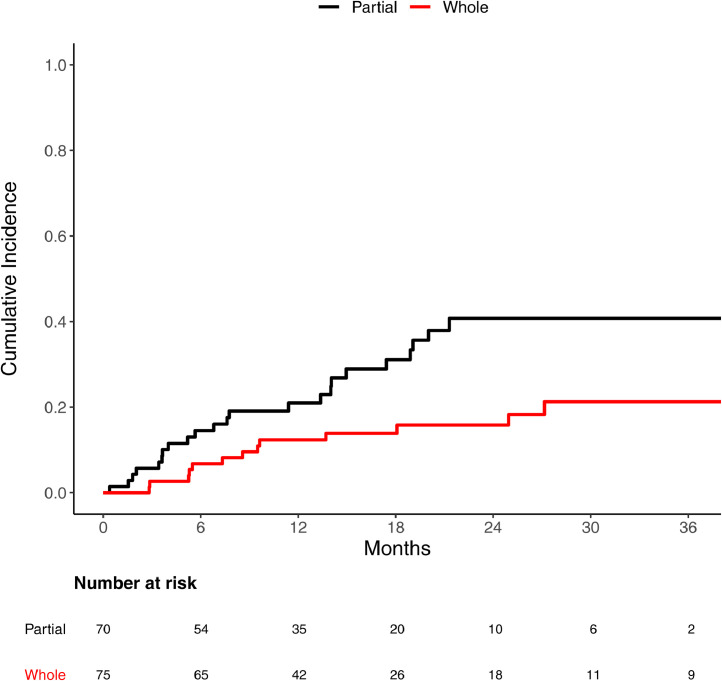
Recurrence coverage. Cumulative incidence of recurrence for patients who had radiation therapy field covering whole hardware or partial hardware.

Figure 1 illustrates the cumulative incidence curve for recurrence between partial and whole coverage. Using univariate Fine-Gray competing risk models, Table 2 shows significant associations with recurrence and whole hardware coverage by RT (0.44 hazard ratio [HR]; 95% CI, 0.22%-0.86; *P* = .017), as well as higher radiation dose (BED10 ≥ median of 39 Gy, 0.5 HR; 95% CI, 0.26%-0.96%; *P* = .038). Intramedullary hardware approached statistical significance (3.29 HR; 95% CI 1%-10.83; *P* = .05).

**Table 2:** Univariate analysis of tumor recurrence

Variable		HR	(95% CI)	*P* value
Age	(y)	0.99	(0.97-1.01)	.3
Sex				.5
	Female	1		
	Male	1.25	(0.66-2.39)	
Primary histology				0.4
	Nonresistant	1		
	Resistant	1.35	(0.76-2.61)	
Time to RT start	(d)	1.01	(0.99-1.02)	.3
KPS				.6
	< Median (80)	1		
	≥ Median (80)	0.83	(0.41-1.68)	
Reaming				.11
	No	1		
	Yes	2.64	(0.8-8.7)	
Translesional				.19
	No	1		
	Yes	1.9	(0.74-4.9)	
Margins				.7
	Positive or none	1		
	Negative	0.79	(0.24-2.6)	
BED10	(Gy)			.038
	< Median (39)	1		
	≥ Median (39)	0.5	(0.26-0.96)	
Hardware				.050
	Plate	1		
	Intramedullary	3.29	(1-10.83)	
Coverage				.017
	Partial	1		
	Whole	0.44	(0.22-0.86)	

*Abbreviations*: BED10 = biologically effective dose (*α*/*β* = 10); CI = confidence interval; HR = hazard ratio; KPS = Karnofsky Performance Status; RT = radiotherapy.

On multivariable analysis including coverage, dosage, and hardware type, none of these factors reached statistical significance (*P = .*19, *P = .*13, and *P = .*15, respectively). The disappearance of significance from univariate analysis to multivariable analysis can occur when there is high correlation among the covariates under investigation. Fisher exact test confirmed an association between coverage and dosage (*P = .*0045), and coverage and hardware type (*P = .*0133), but hardware and dosage were not significantly associated with each other (*P = .*16).

To further test the primary hypothesis given the correlation between covariates, we used propensity score matching, to generate a matched data set (Table 3) of partial versus whole hardware coverage for the following covariates: primary histology, time to RT, KPS, reaming, translesional, margin, BED10, and hardware type. Propensity score matching revealed that whole hardware coverage was statistically associated with a decreased risk of recurrence on the matched dataset (0.24 HR; 95% CI, 0.07%-0.84%; *P = .*026).

**Table 3:** Tumor recurrence propensity score matching

Demographic		Total		Partial		Whole		
		n =	(percent)	n =	(percent)	n =	(percent)	*P* value
Total		70	(100%)	35	(50%)	35	(50%)	
Age*	(y)	62	(52-68)	64	(53-67)	59	(51-68)	.4
Sex								.5
	Female	36	(51%)	16	(46%)	20	(57%)	
	Male	34	(49%)	19	(54%)	15	(43%)	
Primary histology								0.5
	Nonresistant	28	(40%)	12	(34%)	16	(46%)	
	Resistant	42	(60%)	23	(66%)	19	(54%)	
Time to RT start*	(d)	40	(33, 54)	37	(29, 58)	41	(35,48)	.6
KPS								.6
	< Median (80)	23	(33%)	10	(29%)	13	(37%)	
	≥ Median (80)	47	(67%)	25	(71%)	22	(63%)	
Reaming								.5
	Yes	61	(87%)	32	(91%)	29	(83%)	
	No	9	(13%)	3	(9%)	6	(17%)	
Translesional								.8
	Yes	56	(80%)	29	(83%)	27	(77%)	
	No	14	(20%)	6	(17%)	8	(23%)	
Margins								>.9
	Negative	9	(13%)	4	(11%)	5	(14%)	
	Positive or none	61	(87%)	31	(89%)	30	(86%)	
BED10	(Gy)							>.9
	≥ Median (39)	46	(66%)	23	(66%)	23	(66%)	
	< Median (39)	24	(34%)	12	(34%)	12	(34%)	
Hardware								.6
	Intramedullary	55	(79%)	29	(83%)	26	(74%)	
	Plate	15	(21%)	6	(17%)	9	(26%)	

⁎ Statistics presented: median (interquartile range). *Abbreviations*: BED10 = biologically effective dose (*α*/*β* = 10); KPS = Karnofsky Performance Status; RT = radiotherapy.

## Discussion

In this largest study to date analyzing patients with long bone metastases undergoing orthopedic stabilization followed by postoperative RT, we found a significant decrease in local recurrence when the radiation field covers the whole orthopedic hardware. Of note, whole hardware coverage and the other variables included in the multivariate analysis were found to have a high correlation with one another, which led us to perform the propensity score analysis showing a large and statistically significant association between whole hardware coverage and reduced local recurrence.

The finding of reduced local recurrence with whole hardware RT coverage is consistent with other modern retrospective reviews of metastatic recurrence following postoperative conventional RT. Specifically, a study from Harvard found that among 82 solid tumor metastases (including 37% spinal lesions), increasing RT coverage of the hardware (recorded as a continuous variable) was associated with a decreased rate of local failure.^3^ Similarly, a study of 40 lesions from multiple myeloma found a similar reduction in recurrence with increased RT coverage, with an overall recurrence rate of 12.5% at median 25 months of follow-up.^4^ Taken together, these data support the practice of radiation coverage of the whole orthopedic hardware for patients undergoing conventional radiation.

With a median follow-up of 30 months, the cumulative incidence of recurrence in the present study is 30%. This is similar to the prior Harvard study's 17% incidence of recurrence with <12 months once differences between the studies are accounted for, such as the present study's longer follow-up, requirement for surveillance imaging (which likely enriches for recurrence), and inclusion of only nonspinal site of bone metastases.^3^ That study also reported a proportion of in-field recurrence of 71%, which is similar to that found in the present study (75% unadjusted). We found only weak evidence of dose-response relationship on univariate analysis, although this was in the setting of primarily conventional RT and was analysis was limited by larger field size associated with higher BED. Additionally, there was no observable impact of sensitive versus resistant histology on local recurrence. Prior research is somewhat conflicting on the association of dose and recurrence, with some showing no correlation^3^^,^^4^ while others, especially in the setting of spine SBRT, show reduced local recurrence with higher dose.^7^^,^^8^ As patients continue to experience longer survival and use of ablative doses becomes more common in the oligometastatic setting, investigation of novel approaches to radiation in the perioperative setting, such as preoperative ablative radiation, are warranted to potentially further optimize local tumor control.

Use of intramedullary hardware itself was of borderline significance on univariate analysis, and highly correlated with whole field coverage, making it difficult to provide conclusive evidence supporting hardware seeding micro-metastases. Nonetheless, these data continue to generally support this hypothesis. Although one concern with the use of large field radiation might be higher rates of hardware failure, we found that this commonly occurred in the setting of local recurrence (11 of 17). Although RT can lead to hardware failure by impairing bone repair and remodeling,^9^^,^^10^ previous data support the finding that failure is highly correlated with recurrence,^11^ therefore emphasizing tumor control as the primary objective.

This study has several limitations. As in any retrospective analysis of treatment outcomes, confounding by indication could favor the more aggressive treatment, in this case whole hardware RT. Furthermore, high collinearity between both BED10 and hardware type with RT coverage (whole versus partial) limited the multivariable analysis. Although the partial hardware group experienced a larger number of in-field recurrences (n = 17) than the whole hardware group (n = 10), recurrence is a time-to-event outcome and raw proportions of events cannot reliably be compared. Furthermore, the small number of events limits application of inferential statistics. Therefore, although we cannot necessarily attribute the benefit of whole hardware RT to out-of-field recurrence, the finding that whole hardware RT is associated with reduced recurrence is reproducible (including the propensity score matched analysis), and consistent with prior studies. Lastly, the requirement for imaging may bias the sample under study, as there may be more patients lost to follow-up. However, it provides a relatively objectivity definition of recurrence, and all charts were reviewed in duplicate to optimize event capture.

## Conclusions

This study supports the use of whole hardware RT after orthopedic stabilization for solid tumors metastatic to bone in the setting of conventional radiation delivered postoperatively; however, additional data are still needed to elucidate the impact on recurrence of factors including dose, volume, type of surgical hardware used, and sequencing of surgery and RT. The use of 30 Gy in 10 fractions is one appropriate treatment schedule, though datasets including a broader range of doses would help refine an understanding of how treatment schedule affects local recurrence in this setting. An opportunity for clinical trials exists to further improve local control, and might consider investigating optimal sequencing of surgery and radiation.
